# Decreased intrinsic excitability of cerebellar Purkinje cells following optokinetic learning in mice

**DOI:** 10.1186/s13041-020-00678-2

**Published:** 2020-10-07

**Authors:** Yong Gyu Kim, Sang Jeong Kim

**Affiliations:** 1 Department of Physiology, Seoul National University College of Medicine, 103 Daehangno, Jongro-gu, Seoul, 03080 Republic of Korea; 2 Department of Biomedical Sciences, Seoul National University College of Medicine, Seoul, Republic of Korea

**Keywords:** Intrinsic excitability, Cerebellum, Purkinje cell, Oculomotor learning

## Abstract

The optokinetic response (OKR), a reflexive eye movement evoked by a motion of the visual field, is known to adapt its strength to cope with an environmental change throughout life, which is a type of cerebellum-dependent learning. Previous studies suggested that OKR learning induces changes in in-vivo spiking activity and synaptic transmission of the cerebellar Purkinje cell (PC). Despite the recent emphasis on the importance of the intrinsic excitability related to learning and memory, the direct correlation between the intrinsic excitability of PCs and OKR learning has not been tested. In the present study, by utilizing the whole-cell patch-clamp recording, we compared the responses of cerebellar PCs to somatic current injection between the control and learned groups. We found that the neurons from the learned group showed a significant reduction in mean firing rate compared with neurons in the control group. In the analysis of single action potential (AP), we revealed that the rheobase current for the generation of single AP was increased by OKR learning, while AP threshold, AP amplitude, and afterhyperpolarization amplitude were not altered. Taken together, our result suggests that the decrease in the intrinsic excitability was induced in the cerebellar PC of learned group by an increase in the current threshold for generating AP.

## Main text

The optokinetic response (OKR) is a reflex of eye movement that follows the motion of the visual field, which stabilizes an image on the retina. The OKR is exhibited to adapt to changes in the environment throughout life. The cerebellum is well-known to participate in this motor learning as an adaptive controller [1]. The cerebellar Purkinje cell (PC) lies in the center of the adaptive controlling unit. PCs are the sole output of the cerebellum that converge two primary afferent pathways, the parallel fiber (PF) pathway carrying vestibular signals and climbing fiber carrying the error signal in their dendrites. Due to the significance of PCs in neural circuitry modulating OKR, many studies have attempted to find cellular traces of OKR memory in PCs. Early studies performed in vivo unit recording from cerebellar PCs of rabbits in OKR learning, which revealed that spiking activities of PCs were altered after OKR learning in response to optokinetic stimuli [2]. More recently, several studies observed structural and functional changes in PF-PC synapses after OKR learning [3, 4]. However, although it has been suggested that the intrinsic excitability is considered as a crucial cellular correlate of cerebellum-dependent motor learning and memory [5, 6], whether an alteration in the intrinsic excitability is present in PCs following OKR learning has not been verified yet.

Here, we asked whether changes in the intrinsic excitability of PC accompany OKR learning. To address our question, we utilized an ex vivo approach, in which whole-cell patch-clamp recordings were performed from PCs in acute cerebellar slices prepared from mice that underwent OKR learning of 50 min. A similar ex vivo approach has been already taken to elucidate a role of intrinsic excitability in delay eye-blinking conditioning, another form of cerebellum-dependent learning [7, 8].

**Fig. 1:**
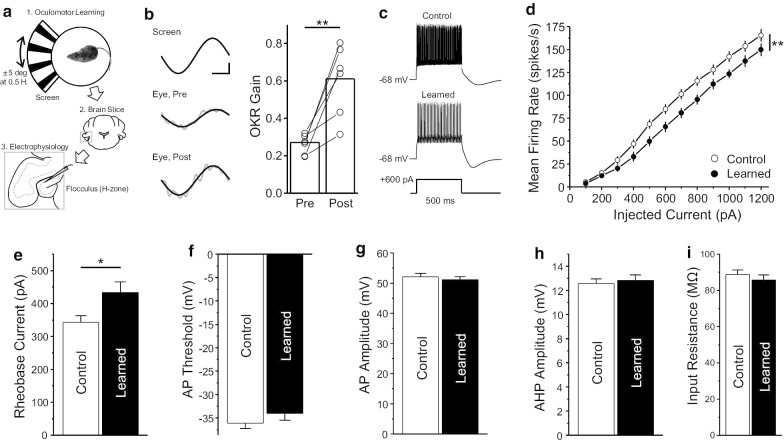
Acute oculomotor learning induces a decrease in the intrinsic excitability of Purkinje cells in the cerebellum. **a** Experimental design. Mice were subjected to oculomotor training for 50 min by rotating an optokinetic screen displaying vertically aligned white and black stripes (top). After the learning, cerebellar slices were prepared (middle) and whole-cell patch-clamp was performed from the floccular PCs located at the H zone (bottom). The dashed line represents the PC layer. **b** (Left) representative traces of the screen, eye movements before (middle) and after (bottom) the learning. In the middle and bottom traces, gray and black lines represent raw and curve-fitted traces, respectively. (Right) Learning-induced increase in OKR gain. A grey circle represents an individual animal (6 mice). Bar graphs indicate the mean values of each time-point. **c** Representative spiking responses in response to depolarizing current injections (bottom) into PCs of the control (top) and learned groups (middle). **d** The mean firing rate significantly decreases in the learned group compared to that of the control group. **e** OKR learning increases the rheobase current. **f** AP threshold, **g** AP amplitude, **h** AHP amplitude, and **i** Input resistance are comparable between the groups. In (**d**–**i**), the control and learned groups are depicted in white and black, respectively. Data represents as mean ± SEM

C57BL/6 mice were subjected to continuous oscillation (0.5 Hz, ± 5° peak-to-peak) of the optokinetic screen for 50 min using the previously described apparatus [9] (Fig. 1a). The OKR gain, a ratio of the eye response to the visual stimulus, was significantly increased after completion of the learning compared with the gain at the beginning (Fig. 1b; t = − 4.51159, df = 5, p < 0.01, paired t-test). After completion of the oculomotor test, we prepared acute coronal cerebellar slices and performed the whole-cell patch-clamp recordings from PCs in the cerebellar flocculus, which is a crucial region for oculomotor learning in the cerebellum [10, 11] (Fig. 1a). All recordings from the flocculus were performed in the midline subregion of the flocculus known to be involved in horizontal eye movement (H zone). We recorded from 89 and 67 floccular PCs in the control (8 mice) and learned (6 mice) groups, respectively. It is known that distinct firing responses existed in cerebellar PCs in response to the somatic current injection in rodents [12]. In this study, an analysis was conducted on regular spiking neurons, the major neuronal population (> 79%) in both groups (Control, 71 neurons; Learned, 53 neurons).

To test the intrinsic excitability of PCs, we injected step currents from + 100 to + 1200 pA (duration of 500 ms with 100 pA increments) into PCs in the presence of NBQX (2,3-dihydroxy-6-nitro-7-sulfamoyl-benzo[f]quinoxaline) and picrotoxin to block excitatory and inhibitory synaptic inputs, respectively (Fig. 1c). Our result revealed that the PCs from the learned group showed a significant reduction in mean firing rate compared with PCs in the control group (Fig. 1d, χ^2^ = 29.97, df = 11, p < 0.01, Linear mixed model post hoc Tukey’s test). To look for changes in properties of the action potential (AP) that could explain the reduction in the excitability of PCs, we performed the single AP analysis in PCs of both groups. For the single AP analysis, we injected a rectangular current step (duration of 50 ms with 10 pA increments) into PCs to evoke a single AP and measured four AP parameters, the rheobase current, AP threshold, AP amplitude, and afterhyperpolarization (AHP) amplitude (see Additional File 1). The rheobase current after oculomotor learning was significantly increased (Fig. 1e; t = − 2.374, df = 91.3, p < 0.05, Two-sample t-test). Unlike the rheobase current, other three properties, AP threshold, AP amplitude and AHP amplitude, were not altered by oculomotor learning (Fig. 1f–h; AP threshold, t = − 1.13, df = 119, p = 0.26; AP amplitude, t = 0.6, df = 118.98, p = 0.55; AHP amplitude, t = − 0.42, df = 119, p = 0.68, Two-sample t-test). Lastly, in response to hyperpolarizing current injection, no difference was found when comparing the input resistance between the groups (Fig. 1i; t = 0.80, df = 120, p = 0.42, Two-sample t-test).

In the present study, we showed a significant decrease in the firing rate of the cerebellar PC in response to the intracellular injection of depolarizing current after 50 min of OKR learning. Further single AP analysis found that the rheobase current was increased in the learned group, while the AP threshold, AP amplitude, and AHP amplitude did not differ significantly between the control and learned groups. Taken our results together, it would be expected that a decrease in the intrinsic excitability was induced in the cerebellar PC of the OKR learning group by an increase in the current threshold for generating AP. Our result supports the emerging hypothesis that cerebellar memory may be implemented not only by a change in synaptic transmission in the Purkinje cell, but also change in the intrinsic excitability [5–8, 13, 14]. Given that the intrinsic excitability of neurons is influenced by the conductance of various transmembrane ion channels that affect the passive and active membrane properties of the neuron, a further attempt should be made to determine the ion conductance that mediates the reduced excitability of cerebellar PCs after OKR learning.

## Supplementary information

**Additional file 1. **Supplementary Methods.

## Data Availability

The datasets used and/or analyzed during the current study are available from the corresponding author on reasonable request. For the analysis of video-oculography and electrophysiological data, custom-built analysis tools, VOG_Analysis_Pack and IntrinsicVIEW, are available in the Github repository, https://github.com/parkgilbong.

## Abbreviations

OKR: Optokinetic response
PC: Purkinje cell
PF: Parallel fiber
AP: Action potential
AHP: Afterhyperpolarization
