# Supplementary material for: Decreased intrinsic excitability of cerebellar Purkinje cells following optokinetic learning in mice
Source: Mol Brain. 2020 Oct 7;13:136. doi: 10.1186/s13041-020-00678-2 (PMC7542746; doi:10.1186/s13041-020-00678-2)
Supplement: Supplementary file 1 — Additional file 1. Supplementary Methods. [file 13041_2020_678_MOESM1_ESM.docx]

**Supplementary Information**

**Decreased Intrinsic Excitability of Cerebellar Purkinje Cells Following Optokinetic Learning in Mice**

Yong Gyu Kim^1,2^ and Sang Jeong Kim^1,2*^

Affiliations

^1^Department of Physiology, Seoul National University College of Medicine, Seoul, Korea

^2^Department of Biomedical Sciences, Seoul National University College of Medicine, Seoul, Korea

^*^Corresponding Author

E-mail address: [sangjkim@snu.ac.kr](mailto:sangjkim@snu.ac.kr)

List of contents

Supplementary Methods

**Supplementary Methods**

***Animal***

 C57BL6/J male mice aged 7- to 9-week-old were used. Animals were housed with food and water available *ad libitum* under a 12 hours light/dark cycle. All animal use was in accordance with protocols approved by the Animal Care and Use Committee of Seoul National University College of Medicine.

***Behavioral test***

*Surgical procedure* The whole surgical process was done under isoflurane anesthesia. In order to restrain the mouse's head during the behavioral test, a headpost was mounted with two M2 nuts, four screws (M1.2 x 5.5), and dental cement (Super bond C&B, Sun Medical, Japan). Nuts were placed approximately on the lambda and bregma of the skull, and screws were implanted beside the nuts. Finally, dental cement was applied between the nuts and screws following the manufacturer’s instruction. Mice were given for at least 24 hours after surgery.

*Apparatus for behavioral tests* Behavioral tests were conducted using custom-made apparatus (Figure 1A). Optokinetic stimulation was generated by a cylindrical screen with vertical black and white stripes. Right eye movement was monitored with a high-speed CCD camera under infrared illumination.

*Learning protocol* Before the start of the learning session, OKR was measured under sinusoidal oscillation of the screen with a rotation amplitude of 5 deg and a rotation frequency of 0.5 Hz. After initial recording, a 50min learning session was run under the same optokinetic stimulation of the initial recording. Immediately after, OKR was measured again.

*Data analysis* The recorded stimulus (movement of the screen) and response (evoked eye movement) were smoothed and fitted to sine curves. Finally, the gain of OKR, a ratio of the eye response to the visual stimulus was obtained. For all computational procedures of the data, we used VOG Analysis Pack, a custom-built video-oculography (VOG) data analysis tool written by LabVIEW (<https://github.com/parkgilbong/VOG_Analysis_Pack>).

***Slice Preparation***

 Mice were anesthetized with isoflurane, then decapitated. Once the brains were extracted, using a vibratome (VT1200, Leica), coronal cerebellar slices (250μm thick) were obtained. The slices were cut in a chamber filled with ice-cold cutting solution, NMDG-HEPES, composed of the following (in mM): 2.5 KCl, 1.25 NaH_2_PO_4_, 93 NMDG, 30 NaHCO_3_, 20 HEPES, 25 glucose, 5 sodium ascorbate, 2 Thiourea, 3 sodium pyruvate, 12 L-acetyl-cysteine, 10 MgSO_4_•7H_2_O and CaCl_2_•2H_2_O bubbled with 95% O2 and 5% CO2. Once the brains were completely sliced, those slices were immediately put into an artificial CSF (ACSF) composed of the following (in mM): 125 NaCl, 2.5 KCl, 1 MgCl_2_, 2 CaCl_2_, 1.25 NaH_2_PO_4_, 26 NaHCO_3_ and 10 glucose bubbled with 95% O2 and 5% CO2. For the recovery, slices were incubated at 32℃ for 15 minutes, and then 1 hour at room temperature.

***Whole-cell patch-clamp recording***

 Slices were placed in a submerged chamber on the stage of a microscope (BX 50WI, Olympus Optical, Japan) and perfused with ACSF. The whole-cell current-clamp recordings were performed from PCs in the cerebellum at 32℃ using the recording patch pipettes (2.5-3.5 MΩ) filled with an internal solution containing the following (in mM): 9 KCl, 10 KOH, 120 K-gluconate, 3.48 MgCl_2_, 10 HEPES, 4 NaCl, 4 Na_2_ATP, 0.4 Na_3_GTP, and 17.5 sucrose, pH 7.25 accompanied with Multiclamp 700B (Molecular Devices) and Digidata 1440A (Molecular Devices). The sampling frequency of 20 kHz and filtering of signals at 2 kHz was kept constant throughout the experiment. All of the recordings were conducted within the ACSF containing 100 μM picrotoxin (Sigma-Aldrich, USA) and 10 μM NBQX (2,3-dihydroxy-6-nitro-7-sulfamoyl-benzo(f)quinoxaline) (Tocris Bioscience, UK) to block inhibitory and excitatory synaptic inputs, respectively. During the current-clamp recording, the membrane potential was maintained at -70 mV. Neurons in which the holding current was exceeded 600 pA were excluded from the analysis.

***Data analysis***

 All whole-cell recording data were analyzed and managed using IntrinsicVIEW Analysis Pack (<https://github.com/parkgilbong/IntrinsicVIEW>). To evaluate the PC excitability, 500 ms-long depolarizing currents were injected into PCs from +100 to +1200 pA with increments of 100 pA. For the analysis of single action potentials (APs), brief depolarizing currents with a duration of 50 msec were injected into PCs with increments of 10 pA until the first AP fires. The rheobase current was defined as the minimum current magnitude required to generate an AP. The voltage threshold of AP was defined by measuring the membrane potential at which its 1st derivatives exceeded 5 mV/ms. The difference between the AP threshold and the positive, and the negative peak of the trace was defined as the AP amplitude and the AHP amplitude, respectively. Input resistance was determined by measuring the difference between the baseline and the maximal negative voltage during hyperpolarizing current injection (from -600 pa to -300 pA with increments of 100 pA) of 500 msec duration.

***Statistics***

 All statistical computing was performed using R (v3.6., <https://www.r-project.org/>). For the assessment of the learning effect in OKR learning, a paired t-test was used. To compare electrophysiological properties between the groups, two-sample t-test and linear mixed model *post-hoc* Tukey test were used for the analysis of single and repeated measurement data, respectively.
